# Manometric Evaluation of the Sphincter Complex in Anterior Anus and Mild Anorectal Malformations—An Important Diagnostic Tool

**DOI:** 10.3390/diagnostics15091078

**Published:** 2025-04-24

**Authors:** Jonathan Hencke, Raphael Staubach, Steffan Loff

**Affiliations:** Department of Pediatric Surgery, Olgahospital, Klinikum Stuttgart, Kriegsbergstrasse 62, 70174 Stuttgart, Germany

**Keywords:** anterior anus, anorectal malformation, anorectal manometry, sphincter complex

## Abstract

**Background:** Distinguishing between the anatomical variant of an anterior anus and mild forms of imperforate anus with rectoperineal fistula often requires inspection, calibration, and, in uncertain cases, electrical stimulation (ES) under general anesthesia. Anorectal manometry (AM), despite its ability to assess sphincter configuration and function, is rarely reported as a diagnostic tool. This study evaluated the utility of AM in such cases. **Methods:** A retrospective analysis of AM and clinical data from 38 patients (35 female, 3 male) with suspected anterior anus was conducted from October 2009 to September 2021. Water-perfused catheter probes with eight radial channels were used to perform pull-through maneuvers. Sphincter locations were identified through vector reconstruction, and pressure ratios of the anterior part to the circumference were recorded. Results were compared to clinical data, including ES findings. Statistical significance was assessed using Mann–Whitney U and Chi-Square tests. **Results:** Following AM, ES was unnecessary in 25 patients. Of the remaining patients, 83% showed abnormal sphincter configurations on ES, and seven underwent anoplasty. Patients with abnormal sphincter complexes demonstrated significantly lower mean anterior pressures (61.2 mmHg vs. 136.4 mmHg, U = 336.5, *p* = 0.001) and a trend toward lower anterior-to-circumferential pressure ratios (mean 0.42 vs. 0.85, U = 613, *p* = 0.270). Constipation was also more frequent in this group (*X*^2^(1,*N* = 38) = 4.1, *p* = 0.044). Average anterior pressures < 75 mmHg and ratios < 0.7 indicated an anus outside the sphincter complex (sensitivity 80%, specificity 90%). **Conclusions:** AM proves valuable for evaluating ambiguous anterior anus cases, potentially reducing reliance on ES under general anesthesia. 3D high-resolution AM may further increase diagnostic accuracy.

## 1. Introduction

In some children, caregivers observe an anteriorized anal opening, often raising concerns about a possible anorectal malformation (ARM). An anus located closer to the genitalia than normal, while still having an average diameter, an adequate perineal body, and surrounded by sphincter muscles, is presently understood as an anatomical variant [1]. Various terms for this are used: ‘anterior anus’ (AA), ‘ectopic anus’ or‚ ‘ventral anal ectopy’, with‚ ‘anterior anus’ being the preferred definition. It must be distinguished from an imperforate anus with a rectoperineal fistula and anal canal stenosis, both considered pathological in contrast [1]. On the one hand, there is a need to identify mild forms of ARM that are sometimes missed but could lead to severe sequelae, especially constipation [2]. On the other hand, it is crucial to avoid misdiagnosing a normally functioning anus and subjecting children to unnecessary surgery. Recently, the ARM-Net Consortium published a comprehensive position paper with clear definitions [1] aiming to standardize the evaluation and treatment of children with an anteriorized anal opening. According to their guidelines, children are categorized as ‘normal anus’ (normal position, normal diameter, surrounded by sphincter muscle), ‘anterior anus’ (anterior position, normal diameter, surrounded by sphincter muscle), ‘congenital anal stenosis’ (normal or anterior position, reduced diameter, surrounded by sphincter muscle), and ‘perineal fistula’ (anterior position, possibly abnormal diameter, not surrounded by sphincter muscle). For differentiation, the authors suggest a systematic approach involving inspection, measuring the position using the ano-perineal index (API), determination of the caliber with Hegar dilators, and elicitation of the anocutaneous reflex to observe sphincter contraction. The API was first proposed by Reisner et al. in 1984 [3] and has become the standard measurement of anal position. It is measured as the ratio of the distance between the scrotum/fourchette and anus and divided by the distance between the scrotum/fourchette and coccyx [3]. An API < 0.34 in girls and <0.46 in boys is considered an anteriorly displaced anus. In uncertain cases concerning the musculature, electrical stimulation (ES) under general anesthesia is the suggested method. Several alternative methods to assess the sphincter have been proposed in the literature. Transperineal sonography [4], endosonography [5], and magnetic resonance imaging [6] have been used with some success.

Manometric evaluation of the sphincter in AA is seldom documented, with only a handful of studies having investigated whether the sphincter muscle surrounds the anal canal [7,8]. In postoperative evaluation of ARMs, however, this is commonly employed to demonstrate sphincter integrity and the ability of the patient to squeeze sufficiently [9]. In our department, we have utilized anorectal manometry (AM) in various investigations, including AA, and thus, we wish to impart our experience in this regard.

## 2. Materials and Methods

**Figure 1:**
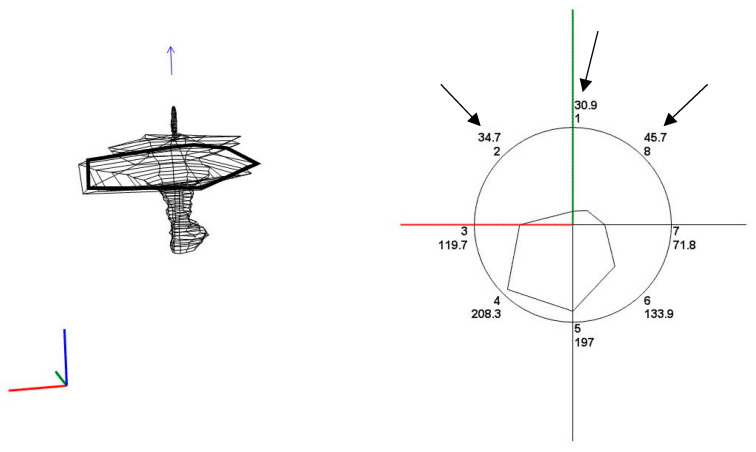
Vector reconstruction of a pull-through maneuver (**left**) with the corresponding transection (**right**); channels 1, 2, and 8 (arrows) are defined as ‘anterior’.

We conducted a retrospective analysis of all patients undergoing AM for suspected AA between October 2009 and September 2021. Each patient received an enema prior to the exam and no sedative was administered. All patients underwent AM with identical equipment: a water-perfused eight-channel manometric catheter with radially placed channels, an outer diameter of 14 Fr, and without a balloon; an electric manometric water pump type PIP-4-8 SS (both Mui Scientific Inc., Mississauga, ON, Canada) with manometric sensors type DPT-6000 (CODAN pvp Critical Care GmbH, Forstinning, Germany) connected to a polygraph (Medtronic, Copenhagen, Denmark) and a computer. Recording and evaluation were performed with the Gastrotrac program (Alpine Biomedical Corp., Fountain Valley, CA, USA). To capture sphincter pressures, the catheter was positioned in the rectum and gradually pulled through the anal canal (at a rate of 1 cm per second) to record the circumferential pressures from the interior towards the exterior of the anal canal. This maneuver was repeated several times. Computerized vector reconstruction subsequently produced a three-dimensional image of the pressures and enabled identification of the high-pressure zone and the sphincter complex, respectively. For this retrospective study, a maximum of six pull-through maneuvers per patient were analyzed and the level with the highest pressure most closely resembling the sphincter complex was examined (Figure 1). All circumferential pressures at this level were recorded; the mean of both the circumference and the three anterior pressures (channels 1, 2, 8; Figure 1) were calculated along with the ratio between these means. As most patients underwent several pull-through maneuvers, we took the minimal, maximal, and mean value of these three measurements (circumferential and anterior pressure, as well as their ratio) in every patient.

Furthermore, we reviewed clinical data from the respective patients, including age, gender, clinical description of AA, whether calibration with Hegar dilators was performed and up to which size, utilization of ES and its result, instances of surgical correction, initial mention of constipation, and any mention of constipation or stool incontinence at follow-up. After data collection, the patients’ information was anonymized for the purpose of analysis. The patients were categorized into two groups: Group A, comprising cases with an abnormal sphincter during ES or a recommendation for ES that was not performed; Group B with a normal sphincter during ES or cases where ES was deemed unnecessary after AM. Statistical analyses were conducted with Microsoft Excel (Version 16.95.1) for means, medians, and other descriptive statistics; the rest were performed with online statistical calculators: sensitivity and specificity calculator from medcalc.org; Mann–Whitney U test and Chi-Square test from soscistatistics.com. Statistical significance was defined as *p* < 0.05. Ethical approval for the retrospective use of patient data without individual consent was obtained from the Institutional Ethics Committee of the University of Heidelberg, Mannheim Medical Faculty, protocol code 2022-821. 

## 3. Results

We identified 38 patients undergoing AM due to clinical suspicion of AA, with the vast majority (35 patients) being female. The age at the initial examination ranged from 35 days to 4 years, with a mean of 8.1 months; the majority (34 patients) were examined in their first year of life. In three cases, AM was repeated at a later stage (ranging from two weeks to twelve months later), either due to inconclusive results at the initial assessment or excessive agitation hindering reliable measurements. Details on constipation, however, were available for all patients, with nine individuals presenting symptoms of constipation at the time of AM.

Regrettably, clinical reports lacked standardization. Descriptions of the anatomy were available for 20 patients, with most cases reporting a small, narrow perineum and measured the perineal length; occasionally, the anal crease, a perineal groove, or the presence of an anal dimple were described. Hegar size was noted in 21 patients; sometimes, the calibration was halted without reaching the maximum possible caliber despite the expected Hegar size being higher. Only one case exhibited a clearly stenotic anal canal with an 8–9 mm diameter. The average diameter across cases was 11.05 mm. The ano-perineal index (API) was not documented for any patient.

The number of pull-through maneuvers per exam ranged from one to ten with an average of 4.5. After AM, further evaluation with ES under general anesthesia was deemed unnecessary in 24 patients. Among the remaining cases, 12 underwent ES with 10 patients (83%) exhibiting an abnormal sphincter complex. In two cases, ES was recommended but not executed (and no surgical intervention occurred). The reports of ES frequently depicted the center of the sphincter complex as being dorsal to the anal opening and noted a complete absence of sphincter muscle on the anterior aspect of the anus. Conversely, the two patients with a normal sphincter during ES either displayed an anus completely encircled by the sphincter or only a slightly weaker anterior part. Group A (abnormal sphincter verified in ES or recommended ES) consisted of 12 patients, while Group B consisted of 26 patients.

**Figure 2:**
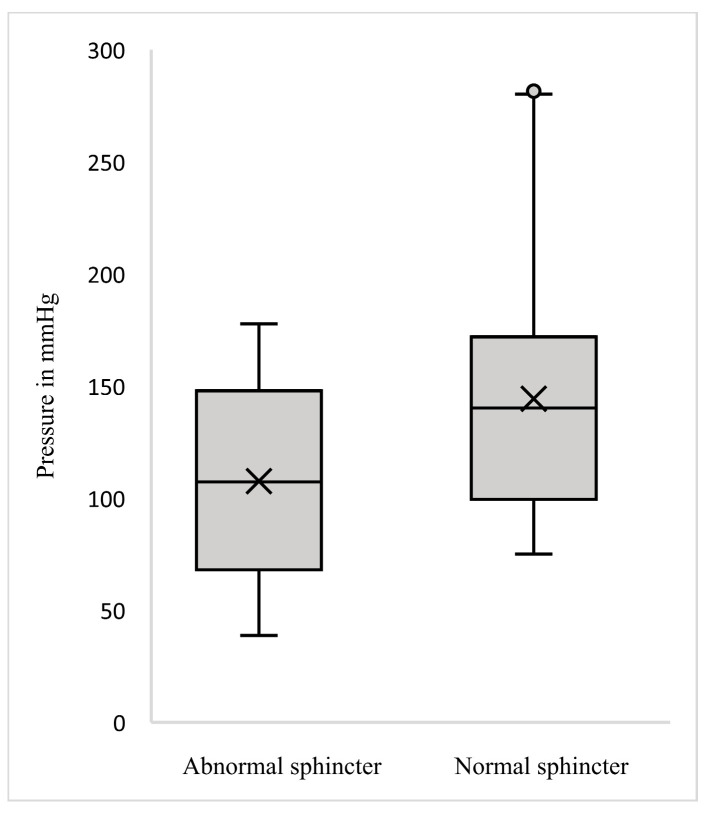
Boxplot of mean circumferential pressures.

**Figure 3:**
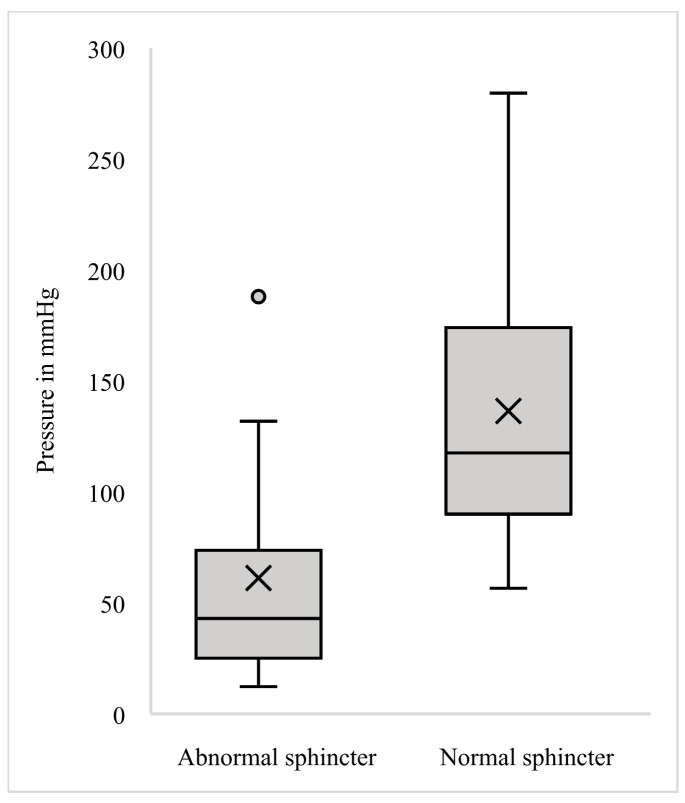
Boxplot of mean anterior pressures.

**Figure 4:**
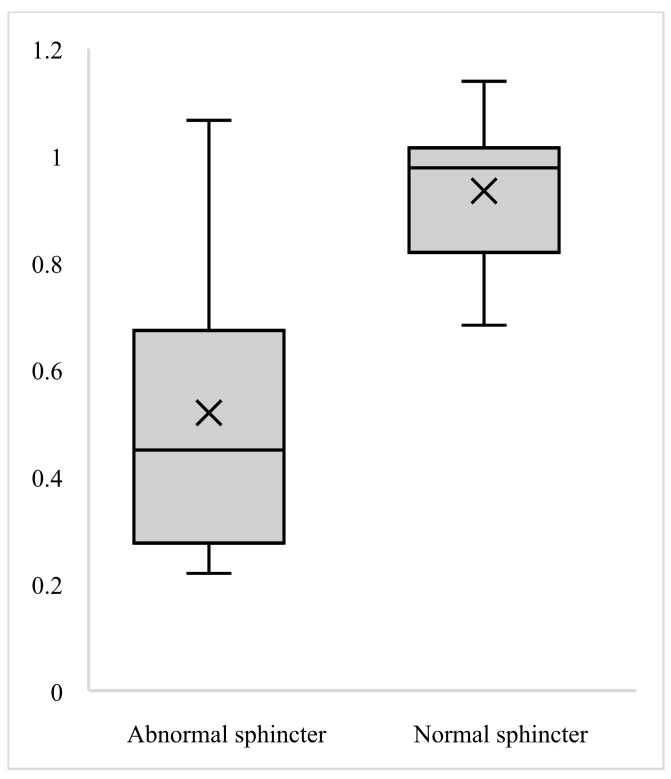
Boxplot of mean ratio anterior-to-circumferential pressures.

We conducted a comparison of circumferential pressures, anterior pressures, and their respective ratio between the two groups; the results are presented in Table 1 and visualized in Figure 2, Figure 3 and Figure 4. Analysis revealed a trend towards lower circumferential pressures as demonstrated in Figure 2. While there was an obvious tendency for lower anterior pressures (Figure 3) and lower ratio of anterior-to-circumferential pressures to be exhibited in Group A (Figure 4), it was only statistically significant for lower anterior pressures (U = 336.5, *p* = 0.001).

**Figure 5:**
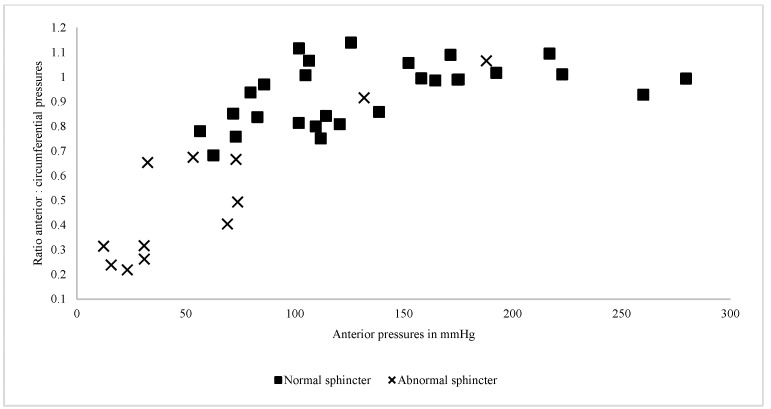
Scatter plot comparing mean anterior pressures and their ratio to circumferential pressures between patients.

As illustrated in Figure 5, there are two outliers, with one of them having a stenotic anal opening. Notably, the majority of cases with an abnormal sphincter were clustered within the area of anterior pressures under 75 mmHg and ratios under 0.7. When establishing these values as a combined cut-off threshold, we reached a sensitivity of 80% (95% CI 44–98%) and a specificity of 90% (95% CI 74–98%) for an abnormal sphincter.

We compared constipation rates between Group A and B, revealing that 45% of Group A patients were reported as constipated at the time of AM while 15% of Group B had a diagnosis of constipation (*X*^2^(1,*N* = 38) = 4.1, *p* = 0.04). Seven patients underwent anoplasty after ES. All patients undergoing surgery presented for follow-up visits, with the most recent visit at a mean age of 5.3 years, range 1.1–12.1 years. Of all remaining patients without surgery, 17 were followed up at a mean age of 4.2 years, range 6 months–13.3 years. No documentation on follow-up visits was found in our hospital records for 14 patients. Two patients undergoing anoplasty were reported as constipated at follow-up, while five were mentioned to be without constipation; when compared with preoperative constipation rates, three experienced an improvement in constipation, two were already without constipation before the procedure, one remained constipated, and one developed constipation after surgery. Most other patients not undergoing anoplasty were without constipation at the time of AM and follow-up (14/17, 82%), with one showcasing improvement at follow-up while one remained constipated; however, the follow-up period in this case was very short (1 month). One patient developed severe constipation, and it is noteworthy that AM had indicated the suspicion of an abnormal sphincter in this patient (mean anterior pressure 73 mmHg and ratio 0.66). Although ES was recommended, the parents opted for further treatment in a different hospital, where bowel management was initiated and a sigmoid resection due to constipation took place; the patient presented her case to a different department of our hospital several years later.

## 4. Discussion

Our results demonstrated a good overlap between findings from clinical, manometric, and electric stimulation data for identifying ARM and AA. This supports the use of AM to evaluate the anal canal in ambiguous cases. Both the statistical significance for lower anterior pressures and the trend towards lower ratios between anterior and circumferential pressures support the presumed findings. The sensitivity and specificity, although not perfect, further bolsters the employment of AM as a promising diagnostic adjunct. Furthermore, the clinical data, with low rates of constipation in Group B (assumed to have a normal sphincter), reinforce the categorization into the groups.

Given the incidence of AA and clinically unremarkable ARM in the general population, our study represents a relatively good number of subjects; however, this was only possible in the form of a retrospective study, which unfortunately lacked standardization and showed inconsistent follow-ups. Especially the missing clinical information on API, Hegar size and description of the severity of constipation or bowel function at presentation or at follow-up in the whole study population would have strengthened the results. There are different definitions and classifications (e.g., Rome IV) of constipation, but we had to rely on the mere mention of constipation in the patient’s record due to incomplete records. Another aspect is an error inherent in AM itself: As most of the children were in their first year of life, no command to squeeze could be given. However, the pull-through maneuvers of the water-perfused catheter usually cause enough anal stimulation to elicit contraction of the sphincter, especially in younger children. The pull-through maneuvers were accomplished by estimating the correct speed (1 cm/s) with the markings on the catheter and a sound at 1 s intervals, but this manual technique is, of course prone, to error. In the same way, not all children were at rest, which might have caused measurement artifacts. Other centers routinely use sedation for AM in children, while we have good experience of reliable measurements without any sedation, especially in the first year of life. The selective utilization of ES only in clinically very suspicious patients and those with conspicuous AM results also undermines the validity of our results. However, this could not be mitigated given the retrospective nature of this study. This study is not free from different sources of bias: there is certainly some selection bias as some patients may have had an almost normal anal position which may not be classified as an AA when measuring the API. AM, the reported incidence of constipation and especially ES can also be prone to considerate observer bias without standardized protocols.

A few studies from various time periods have employed anorectal manometry in AA, with most involving different definitions and techniques, which hampers direct comparison of results: the first study by Kerremans et al. from 1974 utilized microballoons to measure resting pressure and presence of the recto-anal inhibitory reflex (RAIR) [10]; the second study by Schuster et al. from 2000 used the same equipment as our study, focusing on anal canal length and vector volume, as well as sphincter muscle asymmetry in a similar way as in our study [7]; Ruttenstock et al. from 2013 also used water-perfused catheters to evaluate anal canal length, resting pressure, and RAIR in pre- and post-operative cases of ARM [11]. The most recent study from 2023 employed three-dimensional high-resolution AM (3D-HRAM) [8]. However, it is worth noting that all these studies differ in their definition of AA and mild ARM. The older studies used the term ‘ectopic anus’, with most patients suffering from constipation and having undergone some form of anoplasty. Kerremans et al. already acknowledged the possibility of a functionally normal AA without constipation, thus not requiring surgery [10]. The study by Schuster et al. compared children with ‘ectopic anus’ and pre-existing severe constipation to normal children [7]. Some of these children with ‘ectopic anus’ might be more accurately categorized as ARM with a non-stenotic perineal fistula according to contemporary definitions. The correct measurement of anal canal length and the respective vector volumetry, as in the study by Schuster et al. [7], may depend on a very precise and consistent pull of the catheter in manual pull-through maneuvers; therefore, these parameters were not measured in our study.

**Figure 6:**
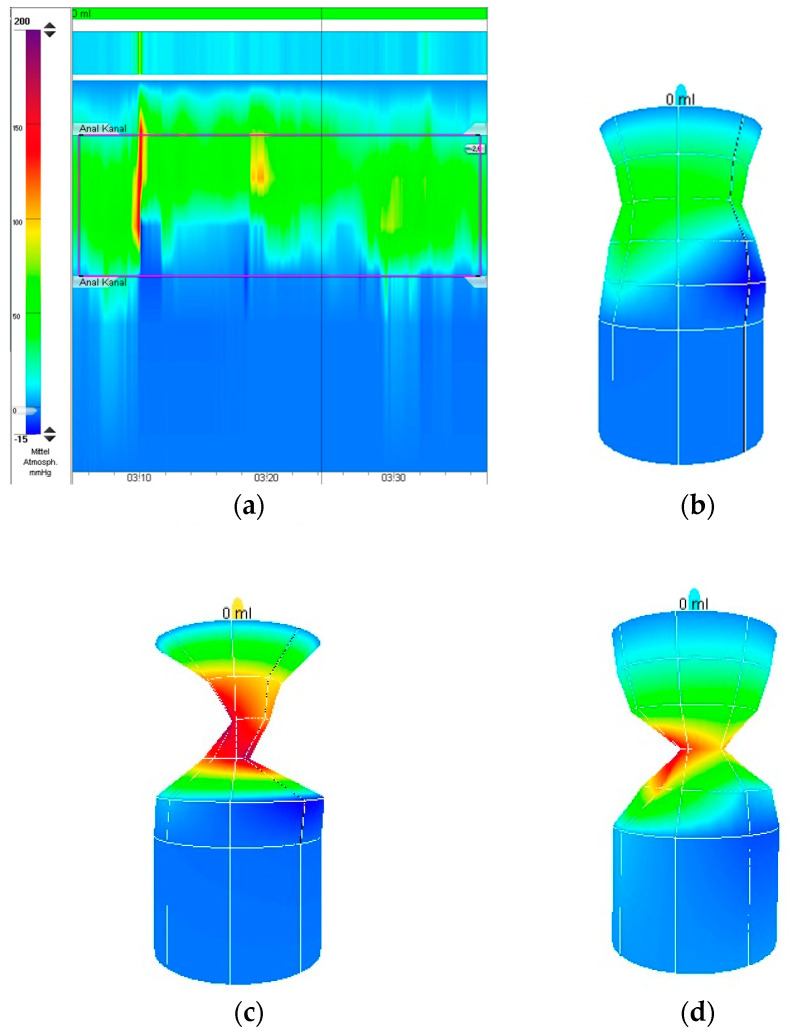
(**a**) 3D-HRAM recording of resting pressure with an occasional squeeze pressure during sneezing; (**b**) 3D reconstruction of AA at rest with circular sphincter complex; (**c**) 3D reconstruction of anal squeeze in the same patient; (**d**) slight anterior weakness during squeeze in a different patient.

The most recent study using 3D-HRAM warrants a special mention: although the study did not adhere to the ARM-Net’s definitions of AA, it demonstrated a significant concordance between 3D-HRAM findings and ES results, comparing children with mild ARM with constipated children and those with Hirschsprung’ disease [8]. The study reported 100% sensitivity and specificity, as ES consistently confirmed the predictions made with 3D-HRAM, particularly if the sphincter was interrupted anteriorly. Since 2022, we transitioned our AM equipment to a solid-state catheter, enabling the application of 3D-HRAM. This should provide an improved image and eliminate the errors caused by the pull-through maneuvers. It is worth noting that normal pressure values should not simply be transferred from studies with water-perfused catheters to the use of solid-state catheters; usually pressures are higher with solid-state sensors [12]. So far, we have examined ten patients with suspected AA with this system with promising results; examples of the recordings and 3D reconstructions are shown in Figure 6a–d. 3D-HRAM provides an excellent image of sphincter function, a feat that is difficult to reproduce with other methods. ES, in comparison, would require video recording to offer a similar objectivity. Since most of our recent patients did not present stenosis or constipation and only a minority showed a slightly weaker anterior sphincter section, only one is planned to be further examined by ES and undergo surgery if ES confirms an anal location clearly outside the sphincter complex.

Adherence to definitions, as advocated by recent guidelines, will help standardize studies, diagnostics, and treatment. Although further studies are necessary, consistent protocols and the increased use of 3D-HRAM are likely to improve diagnostic accuracy. In our current practice, we aim to gather comprehensive data: API, anal caliber, detailed description of outer anal appearance, and clinical symptoms. We encourage our colleagues in the field of pediatric colorectal surgery to adopt this approach and utilize AM, especially 3D-HRAM, if available, for sphincter assessment in order to decrease the need for general anesthesia in ES. Moreover, AM can be readily performed in an outpatient clinic, alleviating concerns for parents who may be apprehensive about their infant receiving general anesthesia; this aspect is also relevant in cases of limited operating room time availability.

The need for surgical treatment in AA and especially in mild ARM remains an ongoing debate. The altered recto-anal angle in AA may impede defecation by creating a rectal ‘cul-de-sac’ and lead to constipation [10]. In the past, constipated cases of AA have been corrected surgically [7,13], while newer studies with relatively large cohorts and long-term follow-up, extending into adolescence and adulthood, have shown that this is mostly unnecessary [14,15]. While constipation was reported up to three times more often in AA than in controls, it remained a minority occurrence (10–36%) and could always be treated conservatively. Additionally, none showed stool incontinence or a higher rate of urinary tract infections. Even stenotic cases could successfully be managed with serial dilations [14]. Hence, surgical correction of the anal position in AA is now considered outdated. Mild forms of ARM with perineal fistula may also sometimes be treated by dilations alone, as some studies show that preserving as much of the anal canal as possible is associated with improved outcomes [11,16]. This should also be incorporated into the surgical approach [17]. Overall, we have to acknowledge that mild types of ARM exist on a spectrum like all other ARM; the transition to AA may not always be clear-cut and some may require a formal surgical correction by anterior or posterior sagittal anorectoplasty more than others.

The establishment of clear definitions (AA vs. ARM with perineal fistula) is a necessary first step. Further research and, for example, a scoring system consisting of anatomic and clinical information are needed to evaluate which ARM patients benefit most from conservative as opposed to surgical treatment.

## Figures and Tables

**Table 1:** Comparison of circumferential and anterior pressures and their ratio between Group A and B.

		Abnormal Sphincter/Group A (12 pat.), Mean Values	Normal Sphincter/Group B (26 pat.), Mean Values	Mann–Whitney U (U=)	*p=*
**Circumferential pressures**	Mean	107.6 mmHg	144.3 mmHg	629.5	0.33
	Min	87.7 mmHg	121.6 mmHg	506.5	0.10
	Max	131.1 mmHg	170.3 mmHg	484.5	0.08
**Anterior pressures in mmHg**	Mean	61.2 mmHg	136.4 mmHg	336.5	0.001
	Min	43 mmHg	113.6 mmHg	384.5	0.03
	Max	83.1 mmHg	162 mmHg	475.5	0.06
**Ratio anterior: circumferential pressures**	Mean	0.52	0.93	633	0.35
	Min	0.42	0.86	613	0.27
	Max	0.65	1.02	603	0.23

## Data Availability

Due to institutional data protection regulations, the data of this study are not publicly available and will only be shared upon reasonable request.

## Abbreviations

The following abbreviations are used in this manuscript:

ARM	Anorectal malformation
AA	Anterior anus
API	Ano-perineal index
ES	Electrical stimulation
AM	Anorectal manometry
RAIR	Recto-anal inhibitory reflex
3D-HRAM	Three-dimensional high-resolution anorectal manometry
